# Structural insights into the potency of SK channel positive modulators

**DOI:** 10.1038/s41598-017-16607-8

**Published:** 2017-12-07

**Authors:** Young-Woo Nam, Razan Orfali, Tingting Liu, Kunqian Yu, Meng Cui, Heike Wulff, Miao Zhang

**Affiliations:** 1 Department of Biomedical and Pharmaceutical Sciences & Structural Biology Research Center, Chapman University School of Pharmacy, Irvine, CA 92618 USA; 2 State Key Laboratory of Drug Research, Shanghai Institute of Materia Medica, Chinese Academy of Sciences, Shanghai, 201203 China; 3 University of Chinese Academy of Sciences, Beijing, 100049 China; 4 Department of Pharmaceutical Sciences, Northeastern University School of Pharmacy, Boston, MA 02115 USA; 5 Department of Pharmacology, School of Medicine, University of California, Davis, CA 95616 USA

## Abstract

Small-conductance Ca^2+^-activated K^+^ (SK) channels play essential roles in the regulation of cellular excitability and have been implicated in neurological and cardiovascular diseases through both animal model studies and human genetic association studies. Over the past two decades, positive modulators of SK channels such as NS309 and 1-EBIO have been developed. Our previous structural studies have identified the binding pocket of 1-EBIO and NS309 that is located at the interface between the channel and calmodulin. In this study, we took advantage of four compounds with potencies varying over three orders of magnitude, including 1-EBIO, NS309, SKS-11 (6-bromo-5-methyl-1*H*-indole-2,3-dione-3-oxime) and SKS-14 (7-fluoro-3-(hydroxyimino)indolin-2-one). A combination of x-ray crystallographic, computational and electrophysiological approaches was utilized to investigate the interactions between the positive modulators and their binding pocket. A strong trend exists between the interaction energy of the compounds within their binding site calculated from the crystal structures, and the potency of these compounds in potentiating the SK2 channel current determined by electrophysiological recordings. Our results further reveal that the difference in potency of the positive modulators in potentiating SK2 channel activity may be attributed primarily to specific electrostatic interactions between the modulators and their binding pocket.

## Introduction

Small-conductance Ca^2+^-activated K^+^ (SK) channels are encoded by the *KCNN* mammalian genes, including *KCNN1* for SK1 (K_Ca_2.1), *KCNN2* for SK2 (K_Ca_2.2), *KCNN3* for SK3 (K_Ca_2.3) and *KCNN4* for SK4 (IK or K_Ca_3.1) channels^1,2^. SK channels resemble voltage-gated potassium channels in their overall tetrameric assembly. There are six transmembrane alpha helical domains that are denoted as S1–S6 in each SK channel subunit. The pore-forming P-loop between the transmembrane S5 and S6 domains is responsible for the potassium ion selectivity. Both the amino- and carboxyl- termini of these channel proteins are on the intracellular side of the plasma membrane. In the proximal carboxyl-terminus of the channels, there is a region called calmodulin binding domain (CaMBD). The Ca^2+^ binding protein calmodulin (CaM) that is constitutively associated at the CaMBD serves as the Ca^2+^ sensor of SK channels^3^. In response to physiological signals, elevated intracellular Ca^2+^ levels enable Ca^2+^ binding to CaM, inducing conformational changes in the channel carboxyl- terminus and subsequently opening the channel pore^4–6^. As such SK channels are not voltage-dependent but instead rely solely on the Ca^2+^/CaM mediated mechanism described above for their gating. SK channels are therefore often located in close proximity to Ca^2+^ conducting channels or intracellular Ca^2+^ sources^1,2^.

SK channels play essential roles in the regulation of membrane excitability by Ca^2+^ in both the central nervous and cardiovascular systems^1,2^. In the cardiovascular system, SK channels in the heart have been shown to contribute to the regulation of the cardiac action potential^7–11^ while SK channels in the endothelium are implicated in the regulation of vascular tone^12–15^. In the central nervous system, activation of SK channels generates the medium afterhyperpolarization (mAHP) and reduces the firing frequency of action potentials, thus contributing to the regulation of neuronal excitability^1,16^. In neurons, SK channels are subject to regulation by posttranslational modification; e.g., phosphorylation. Ca^2+^ sensitivity of SK channels is reduced by phosphorylation of a specific threonine residue (Thr79) in CaM. The phosphorylation status of CaM Thr79, and thus Ca^2+^ sensitivity of the channels, is regulated by an interplay between casein kinase 2 and protein phosphatase 2A^2,17^. For example, neurotransmitters such as acetylcholine^18^ and norepinephrine^19^ can induce changes in the phosphorylation status of CaM Thr79, and thus regulate the Ca^2+^ sensitivity of SK channels and neuronal excitability.

The pharmacology of the SK channels is relatively well developed. Apamin, previously considered a blocker, actually acts on the SK channel outer pore through an allosteric mechanism^20,21^. TRAM-34 in contrast is a potent inner pore blocker of the IK channel^22,23^, while UCL-1684 and its analogs bind to the outer vestibule of the SK channels^24^. Negative modulation of SK1-3 channel subtypes can be achieved by NS8593, a compound that apparently reduces the Ca^2+^ sensitivity of the channels^25,26^. Interestingly, the negative gating modulator BU-TRMF exhibits selectivity of the SK1 channel^27^, whereas RA-2 is a negative modulator for SK3 and IK channel subtypes^28^. On the other hand, positive modulators of SK channels such as 1-ethyl-2-benzimidazolinone (1-EBIO)^29^ and NS309^30^ can potentiate SK channel activity.

Given the essential roles of SK channels in the cardiovascular system and the central nervous system, SK channels have been proposed as drug targets for the treatment of hypertension^31–35^ and movement disorders^36–41^. Purkinje cells in the cerebellum are the primary locus of pathology in spinocerebellar ataxia^42,43^. SK channel positive modulators have been demonstrated to normalize firing rates of cerebellar Purkinje cells through SK2 channel activation and to exert beneficial effects in mouse models of ataxia^40,44,45^. Accordingly a significant amount of effort has been devoted to developing small molecules that target SK channels^46^. The prototype 1-EBIO was identified almost two decades ago as a positive modulator^29^. 1-EBIO increases the Ca^2+^ sensitivity of SK channels, which enhances the mAHP and effectively modulates neuronal excitability^47^. A Scandinavian biopharmaceutical company (NeuroSearch A/S) has developed additional compounds, including NS309^30^.

Despite all this progress, it remained unknown where on the SK channels these positive modulators bind, until our previous publications^48,49^. We first discovered the binding site of 1-EBIO at the CaM-channel interface. This was done using a combination of approaches, including x-ray crystallography, computational biology and electrophysiology. In the crystal structure (PDB Code: 4G28), 1-EBIO bound at the interface of CaMBD and the CaM N-lobe, forming close contacts with both CaM and CaMBD. Later we found that the more potent SK channel positive modulator NS309 also binds into the same binding pocket as 1-EBIO (PDB Code: 4J9Z).

Here we describe a trend between the interaction energy (E_int_) of the compounds within their binding site calculated from the crystal structures, and the potency (EC_50_) of these compounds in potentiating the SK2 channel current determined by electrophysiological recordings. This was done utilizing four compounds,1-EBIO, NS309, SKS-11 (6-bromo-5-methyl-1*H*-indole-2,3-dione-3-oxime) and SKS-14 (7-fluoro-3-(hydroxyimino)indolin-2-one), varying up to 1000-fold in potency. We investigated the activity of the compounds through electrophysiology and the binding modes by determining x-ray crystal structures of the modulators bound within their binding pocket. Further analysis of the crystal structures show that the strength of electrostatic interactions may be the primary reason why some compounds are more potent than others. Our results provide structural insights into how the potency of SK channel modulators can be improved and thus may facilitate the future development of more potent modulators.

## Results and Discussion

### The potency of SK channel positive modulators

**Figure 1:**
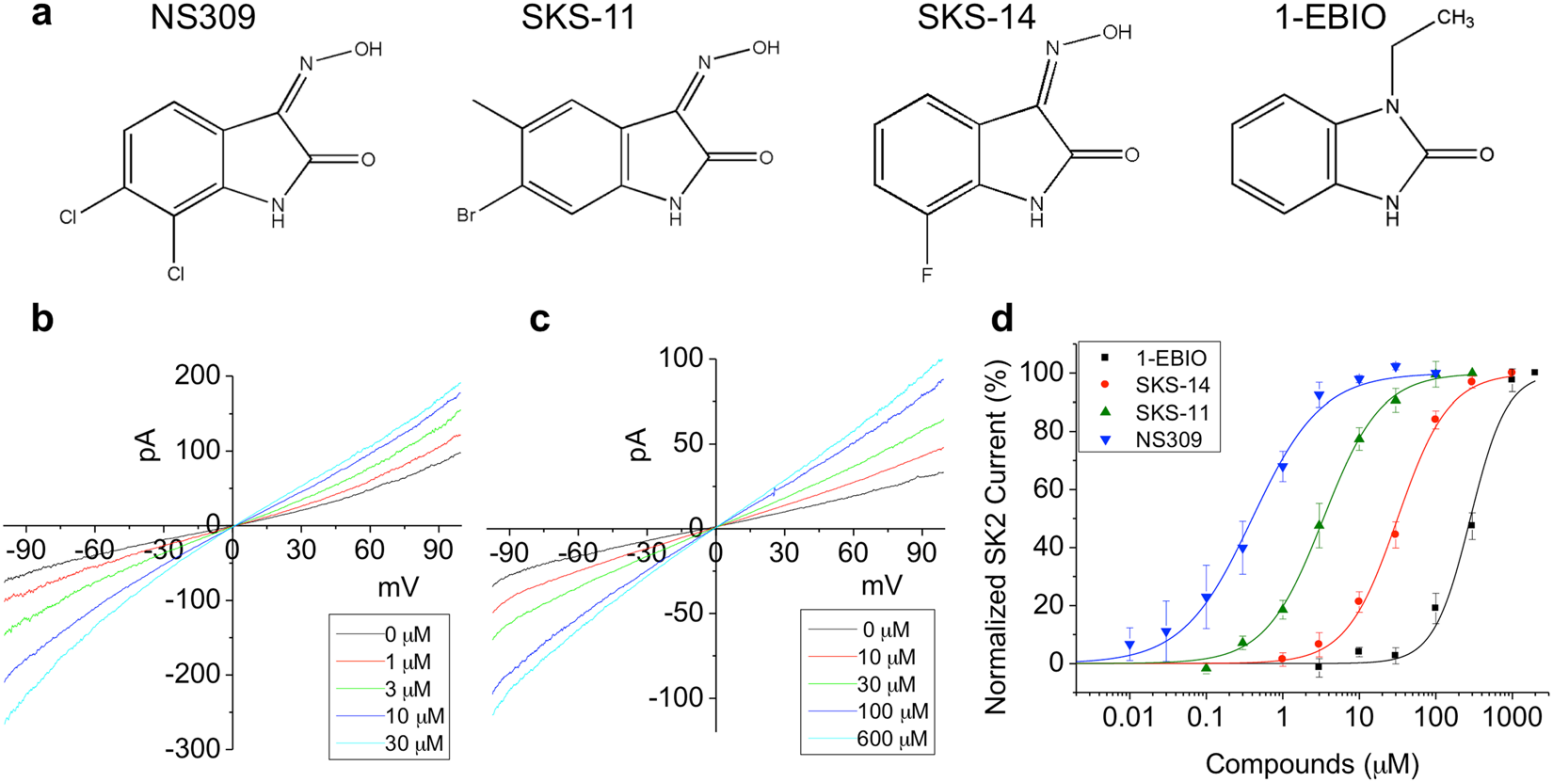
SK channel positive modulators. (**a**) The chemical structures of NS309, 1-EBIO, SKS-11 and SKS-14. (**b**) Raw current traces of dose-dependent channel potentiation by SKS-11 in the presence of 0.1 μM Ca^2+^. (**c**) Raw current traces of dose-dependent channel potentiation by SKS-14 in the presence of 0.1 μM Ca^2+^. (**d**) Dose-dependent potentiation of the SK2 current by NS309, SKS-11, SKS-14 and 1-EBIO, respectively. All data are presented in mean ± s.e.m.

Previously we discovered a binding pocket in the SK2 channel shared by two positive modulators 1-EBIO and NS309^48,49^. In this study, we utilized the crystal structure of the NS309 molecule in complex with its SK2 binding pocket (PDB code: 4J9Z) for computer based virtual screening. The Glide module in Maestro^50^ was used for virtual screening of the Specs library (www.specs.net), which yielded a list of 30 hit compounds. These compounds were purchased and their effects on the SK2 current were tested using inside-out patch-clamp recordings as previous described^48,49,51^. Positive modulators of SK channels like 1-EBIO and NS309 (Fig. 1a) increase the SK channel activity at Ca^2+^ concentrations below the EC_50_ for Ca^2+^ activation of these channels^16,30^. The EC_50_ for the activation of SK2-a channel by Ca^2+^ is ~0.32 μM in our previous report^6^. Therefore we tested these compounds up to the concentration of 1 mM in the presence of 0.1 μM Ca^2+^. Two compounds (SKS-11 and SKS-14, Fig. 1a) potentiated the SK2 current (Figs 1b and c). The other 28 compounds did not induce any change in the SK2 current. The chemical structures and Specs ID (www.specs.net) are shown in Supplementary Fig. S1.

The two positive modulators SKS-11 and SKS-14 closely resemble 1-EBIO and NS309 in their chemical structures (Fig. 1a). Similar to 1-EBIO and NS309, both SKS-11 and SKS-14 dose-dependently potentiated SK2 channel activity at 0.1 μM Ca^2+^ (Figs 1b and c). These two positive modulators demonstrated similar efficacy compared to 1-EBIO and NS309 in potentiation of SK2 channels (Supplementary Fig. S2). The maximal responses to the modulators were normalized by the SK2 currents induced by the saturating Ca^2+^ concentration of 10 μM. The normalized maximal responses to NS309, SKS-11, SKS-14 and 1-EBIO were 93.02 ± 2.85% (n = 8), 94.60 ± 3.03% (n = 8), 92.03 ± 3.44% (n = 9) and 96.17 ± 4.20% (n = 6) of the currents induced by 10 μM Ca^2+^, respectively (Supplementary Fig. S2). There is no significant difference between the normalized maximal responses of these compounds.

On the other hand, these four modulators differed in their potency (EC_50_ values) from each other (Supplementary Fig. S2). The concentration-response curves of these modulators are shown in Fig. 1d. The EC_50_ values of NS309, SKS-11, SKS-14 and 1-EBIO for potentiating SK2 current are 0.552 ± 0.083 μM (n = 8), 3.87 ± 0.88 μM (n = 8), 33.2 ± 4.8 μM (n = 9) and 286 ± 31 μM (n = 6) respectively. NS309 is about 500-fold more potent than 1-EBIO. The potency of SKS-11 and SKS-14 falls between 1-EBIO and NS309. To confirm that the currents that we measured indeed came from the SK2 channel, we utilized apamin. Apamin (20 nM) almost completely abolished the current measured in control experiments (Supplementary Fig. S3).

A moiety shared by the four active compounds is a benzene ring fused with a heterocyclic five-member ring containing at least one nitrogen atom (Fig. 1a). The substituents on the benzene ring include electron withdrawing halogen groups (chloro, bromo and fluoro) and an electron donating methyl group. Fluoro substitution at position 7 (SKS-14), bromo substitution at position 6 (SKS-11) or chloro groups at both position 6 and 7 (NS309) might direct the distribution of electron density from the neighboring heterocyclic ring. It seems that the compounds with halogen groups at the position 6 and 7 are more potent in potentiating the SK2 channel than compounds lacking halogen substituents in these positions (Fig. 1d).

### The shared binding pocket of SK channel positive modulators

We performed virtual screening hoping to discover new scaffolds different from NS309 and 1-EBIO. However, we were only able to identify two active compounds, SKS-11 and SKS-14, which both closely resemble 1-EBIO and NS309 in their chemical structure (Fig. 1a). In future studies, the model needs to be parameterized and trained better to allow for the identification of new scaffolds different from NS309 and 1-EBIO. One possible approach is to utilize multiple conformations of the binding pocket generated from Molecular Dynamics simulations for virtual screening to discover new scaffolds.

**Figure 2:**
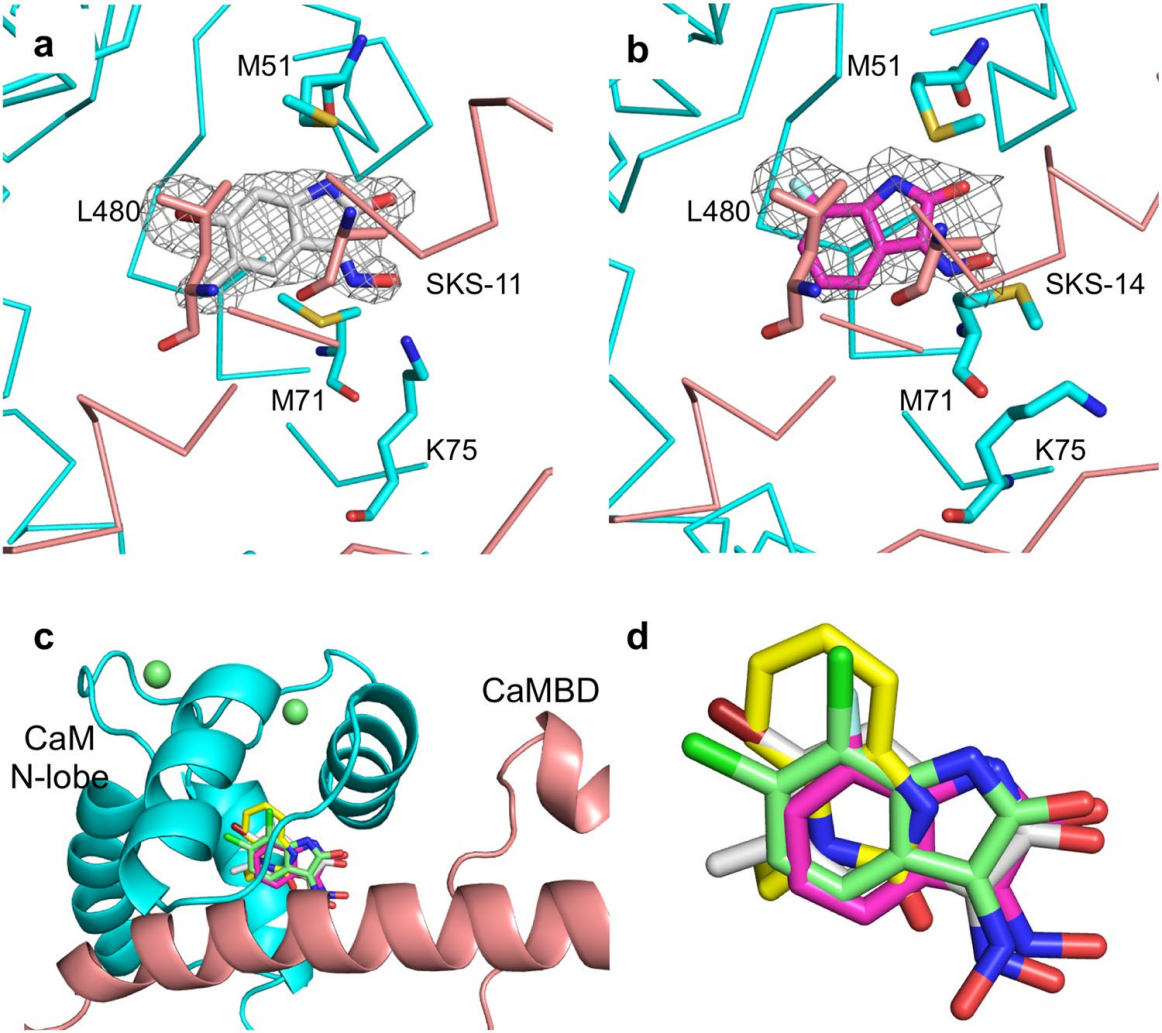
Shared binding site of the SK channel positive modulators. (**a**) Electron density map (grey, |*F*_o_|-|*F*_c_|) showing the presence of additional electron density at the interface between CaM (cyan) and the CaMBD (salmon). The map is contoured at 3.7 σ and is overlaid with the current refined coordinates for SKS-11. (**b**) Electron density map (grey) showing the presence of additional electron density. The map is contoured at 3.7 σ and is overlaid with the current refined coordinates for SKS-14. (**c**) NS309, SKS-11, SKS-14 and 1-EBIO all bind to the interface between the CaM (cyan) N-lobe and the CaMBD (salmon). (**d**) Overlaid conformations of compounds obtained from their respective protein complex crystal structures. The carbon atoms of the compounds are shown in pale green (NS309), grey (SKS-11), magenta (SKS-14) and yellow (1-EBIO).

The identification of SKS-11 and SKS-14 that closely resemble 1-EBIO and NS309 in structure, however, offers us an opportunity to correlate structure and activity at the atomistic level. To address this question, we first sought to determine the crystal structures of SKS-11 and SKS-14 in complex with their binding pocket (PDB codes, 5WBX and 5WC5). Reminiscent of the binding pockets of 1-EBIO and NS309^48,49^, the binding site for SKS-11 and SKS-14 is also located at the interface of CaMBD and the CaM N-lobe (Fig. 2a and b). At the CaM-channel interface, between M51, M71, K75 of CaM and L480 of CaMBD, there is extra electron density apparently coming from SKS-11 and SKS-14, respectively. Overlaid conformations of compounds obtained from their respective protein complex structure show subtle but clear differences, especially for 1-EBIO (Fig. 2c and d). Nonetheless, these crystal structures demonstrate that NS309, SKS-11, SKS-14 and 1-EBIO all bind to a shared binding pocket at the CaM-channel interface.

### The interaction energy of modulators within the binding pocket

After obtaining crystal structures (Fig. 2) of the binding pocket in complex with compounds of different potency (Fig. 1d and Supplementary Fig. S2), we next explored the interactions between the binding pocket and the compounds and correlated them to the compound potency.

**Figure 3:**
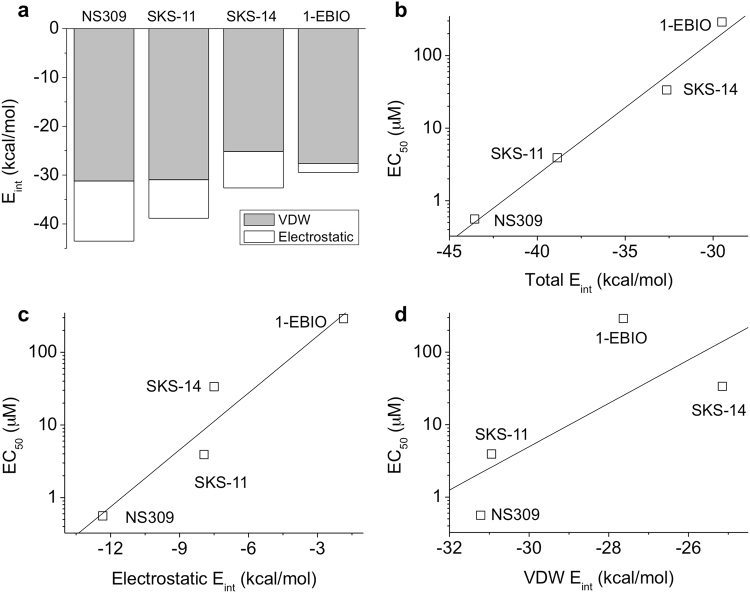
E_int_ between the compounds and their binding pocket. (**a**) Stacked column plot showing the total E_int_ that is composed of VDW E_int_ (grey) and electrostatic E_int_ (white). (**b**) The correlation between the total E_int_ and the potency of NS309, SKS-11, SKS-14 and 1-EBIO (r = 0.99). (**c**) The correlation between the electrostatic E_int_ and the potency of NS309, SKS-11, SKS-14 and 1-EBIO (r = 0.91). (**d**) The lack of correlation between the VDW E_int_ and the potency of NS309, SKS-11, SKS-14 and 1-EBIO (r = 0.74). In (**b,c** and **d)**, the y-axis is set in log-10 scale.

NS309, SKS-11, SKS-14 and 1-EBIO all primarily form contacts with L480 of the CaMBD and with M51, M71 and K75 of CaM. Motivated by the different conformations of the compounds in the binding pocket (Fig. 2), we calculated the interaction energy (E_int_) between the compounds and the binding pocket from the crystal structures using Discovery Studio 3.5 molecular modeling program (Accelrys Software Inc.). The total E_int_ is −43.5 kcal/mol for NS309, −38.8 kcal/mol for SKS-11, −32.6 kcal/mol for SKS-14 and −29.5 kcal/mol for 1-EBIO (Fig. 3a). The total E_int_, calculated from the structures of the compound-bound complexes correlates extremely well with the potency (EC_50_) of the compounds (r = 0.99, Fig. 3b).

In the Discovery Studio 3.5 molecular modeling program, which is using the CHARMM force field, the total interaction energy is defined as the sum of the Van der Waals force (VDW, e.g. repulsion or attraction between atoms) and electrostatic interactions (e.g. interactions due to distribution of the electrons including hydrogen bonds) (Fig. 3a). The interface between CaM and its target protein is relatively hydrophobic^4,6,52^. It is therefore not surprising that the VDW E_int_ contributes to a big portion of the total E_int_ between these compounds and their largely hydrophobic binding pocket. The VDW E_int_ is −31.2 kcal/mol for NS309, −30.9 kcal/mol for SKS-11, −25.1 kcal/mol for SKS-14 and −27.6 kcal/mol for 1-EBIO (Fig. 3a). On the other hand, the electrostatic E_int_ is −12.3 kcal/mol for NS309, −7.9 kcal/mol for SKS-11, −7.5 kcal/mol for SKS-14 and −1.8 kcal/mol for 1-EBIO (Fig. 3a). The potency of the four compounds is much better correlated with the electrostatic E_int_ (r = 0.91, Fig. 3c) than the VDW E_int_ (r = 0.74, Fig. 3d). As such, even though the VDW E_int_ contributes a bigger portion to the total E_int_, the electrostatic E_int_ is actually the primary reason why these compounds exhibit potency differences.

### The electrostatic interactions of modulators within the binding pocket

**Figure 4:**
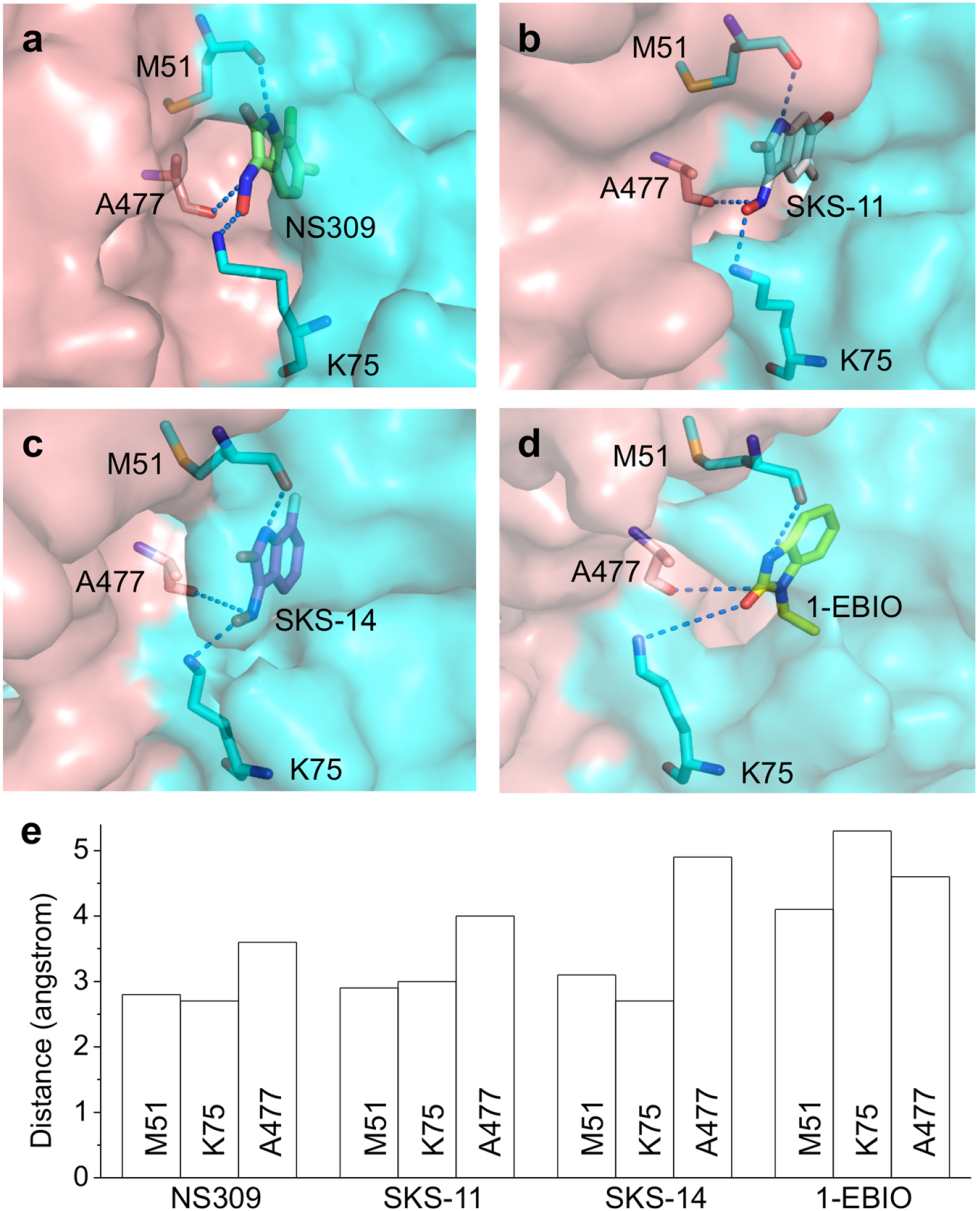
Electrostatic interactions between the compounds and their binding pocket. The amino acid residues M51, K75 and A477 within the binding pocket are shown in the crystal structures in complex with (**a**), NS309 (PDB code, 4J9Z) (**b**), SKS-11 (PDB code, 5WBX) (**c**), SKS-14 (PDB code, 5WC5) (**d**), 1-EBIO (PDB code, 4G28), respectively. (**e**) The distances between the main chain oxygen of M51, the main chain oxygen of A477, the side chain nitrogen of K75 and the compounds.

Due to the importance of the electrostatic E_int_ for the potency differences of the four compounds, we examined the amino acid residues within the binding pocket that form electrostatic interactions with the compounds, namely M51 and K75 from CaM together with A477 from the CaMBD. The oxygen and nitrogen atoms in NS309 (Fig. 4a), SKS-11 (Fig. 4b), SKS-14 (Fig. 4c) and 1-EBIO (Fig. 4d) that form electrostatic interactions are connected by dashed lines with these three residues in the crystal structures. Generally, the distance from these residues is shorter for the more potent compounds (NS309 < SKS-11 < SKS-14 < 1-EBIO, Fig. 4e).

For instance, the distance and angles (θ) from the main chain oxygen of M51 to the nitrogen in the five-member heterocyclic ring is 2.8 angstrom (θ = 134.7°) for NS309, 2.9 angstrom (θ = 164.2°) for SKS-11, 3.1 angstrom (θ = 129.7°) for SKS-14 and 4.1 angstrom (θ = 120.6°) for 1-EBIO. The distance from the main chain oxygen of A477 to the nitrogen in the “ = N-OH” group is 3.6 angstrom (θ = 129.9°) for NS309, 4.0 angstrom (θ = 105.2°) for SKS-11 and 4.9 angstrom (θ = 92.8°) for SKS-14. There is no the “=N-OH” group in 1-EBIO (Fig. 1a). The main chain oxygen of A477 may interact weakly with the other nitrogen in the five-member ring with a distance of 4.6 angstrom (θ = 90.9°). The distance from the side chain nitrogen (Nζ) of K75 to the oxygen in the “=N-OH” group is 2.7 angstrom (θ = 93.3°) for NS309, 3.0 angstrom (θ = 135.8°) for SKS-11 and 2.7 angstrom (θ = 102.7°) for SKS-14. 1-EBIO that lacks the “=N-OH” group, may undergo a weak interaction with the side chain nitrogen of K75 through its “=O” group with a distance of 5.3 angstrom.

**Figure 5:**
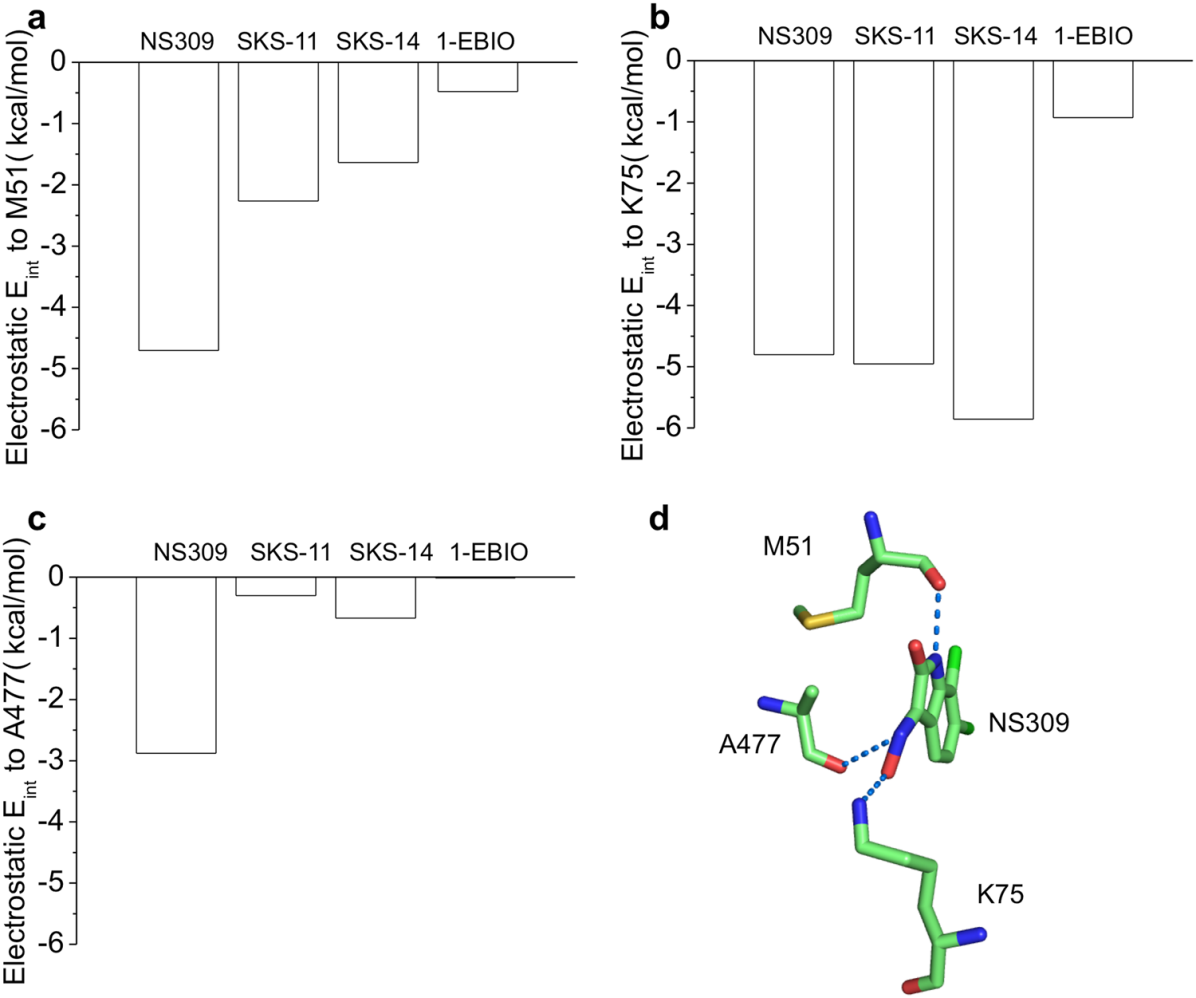
Electrostatic E_int_ between the compounds and amino acid residues in their binding pocket. (**a**) The electrostatic E_int_ between the compounds and M51. (**b**) The electrostatic E_int_ between the compounds and K75. (**c**) The electrostatic E_int_ between the compounds and A477. (**d**) NS309 forms strong electrostatic interactions with three residues M51, K75 and A477.

As electrostatic E_int_ (e.g. hydrogen bond) is closely related to the length and angle of the interaction, we further examined the electrostatic E_int_ between the compounds and the three residues. Consistent with our findings from the crystal structures (Fig. 4), M51 and K75 of CaM together with A477 of the CaMBD are the residues that form electrostatic interactions stronger than −1 kcal/mol with the compounds. The electrostatic E_int_ with the M51 is −4.7 kcal/mol for NS309, −2.3 kcal/mol for SKS-11, −1.6 kcal/mol for SKS-14 and −0.5 kcal/mol for 1-EBIO (Fig. 5a). The electrostatic E_int_ with the K75 is −4.8 kcal/mol for NS309, −4.9 kcal/mol for SKS-11, −5.9 kcal/mol for SKS-14 and −0.9 kcal/mol for 1-EBIO (Fig. 5b). The electrostatic E_int_ with the A477 is −2.9 kcal/mol for NS309, −0.3 kcal/mol for SKS-11, −0.7 kcal/mol for SKS-14 and −0.01 kcal/mol for 1-EBIO (Fig. 5c). NS309 is the only compound that forms electrostatic interactions with all three residues with electrostatic E_int_ stronger than −1 kcal/mol (Fig. 5d), which might explain its high potency in potentiating the SK2 channel (Fig. 1d **)**. Compared to the 6-bromo atom of SKS-11 and 7-fluoro atom of SKS-14, the 6,7-chloro atoms on the benzene ring of NS309 might have enabled optimized the electrostatic interactions between NS309 and the residues of M51, K75 and A477.

Electrostatic interactions such as hydrogen bonds are important specific interactions between ligands and the protein structures, which are often used to improve the efficacy of small molecule drugs through structure-aided drug design^53,54^. Our results here show that the potency of a series of NS309 analogs correlates extremely well with their E_int_, suggesting that one strategy to further improve the potency of SK channel modulators could be to further increase the strength of electrostatic interactions when searching for novel compounds through structure-aided drug design.

## Method

### Virtual Screening

The crystal structure of the NS309 molecule in complex with its binding pocket in the CaM/CaMBD complex from the SK2-a channel (PDB code: 4J9Z) was prepared with the workflow of Protein Preparation Wizard implemented in Maestro. Hydrogen atoms were added, followed by optimization of hydrogen bonds. Then energy was optimized to refine improper atoms using the OPLS2005 force field. Afterwards, the generation of a receptor grid was performed with Glide^50^ in Maestro. The location of NS309 was used as ligand-binding site. A box with a length of 12 angstroms, which surrounded the ligand-binding site, was set for grid generating.

The Specs library that contains approximately 200,000 compounds was used as the ligand database (www.specs.net). These ligands were filtered with Pipeline Pilot (version 7.5)^55^ in the following successive steps. The organic filter and knowledge-based HTS filter were applied for removing inorganic, undesirable and potentially toxic molecules. Then, the application of the Lipinski’s rule of five^56^ removed structures unlikely to have drug-like properties. Subsequently, PAINS (pan-assay interference compounds)^57,58^ were removed. The remaining 179,098 compounds were prepared with LigPrep implemented in Maestro^59^, generating possible protonation states and different stereoisomers using Epik at the target pH of 7.0.

The Glide^50^ module in Maestro was used for virtual screening. The prepared ligands were docked into the binding pocket with the Standard Precision (SP) mode. During the docking process, dock flexible parameters were selected. The resulting poses were ranked by their glide docking scores. Meanwhile, the NS309 molecule was extracted from the complex structure, prepared, and was docked into the structure with the same parameters, scoring -7.780 in SP mode. In the virtual screening, a total of 10,242 poses scored less than -7 were obtained, which were subsequently redocked into the structure with Extra Precision (XP) mode. The top-ranked 2000 poses were first filtered by removing duplicate molecules. We then clustered the molecules into clusters using fingerprint similarity evaluation in chemical, followed by visual inspection to check the interaction mode for every pose in the complex system (small molecule docking with protein), selecting the rational poses from different clusters. Finally, 30 compounds were purchased from Specs (www.specs.org) for further testing by electrophysiological recordings. Two of them (SKS-11 and SKS-14) potentiated the SK2 current. The remaining 28 compounds did not change the SK2 current. The control compounds NS309 and 1-EBIO were purchased from Tocris. Apamin was purchased from Alomone Labs.

## Electrophysiology

The electrophysiological recordings of SK2 current were performed as described in our previous papers^48,49,51^. Briefly, SK2-a channels, along with CaM and GFP, at a ratio of 5:2.5:1 (weight), were transfected into TsA201 cells by the calcium–phosphate method. SK2 currents were recorded 1–2 days after transfection, with an Axon200B amplifier (Molecular Devices) at room temperature.

pClamp 10.5 (Molecular Devices) was used for data acquisition and analysis. The resistance of the patch electrodes ranged from 3–5 MΩ. The pipette solution contains (in mM): 140 KCl, 10 Hepes (pH 7.4), 1 MgSO_4_. The bath solution containing (in mM): 140 KCl, 10 Hepes (pH 7.2), 1 EGTA, 0.1 Dibromo-BAPTA, and 1 HEDTA was mixed with Ca^2+^ to obtain the desired free Ca^2+^ concentrations, calculated using the software by Chris Patton of Stanford University (http://www.stanford.edu/~cpatton/maxc.html). The Ca^2+^ concentrations were verified using Fluo-4 and standard Ca^2+^ buffers (Thermo Fisher Scientific).

Currents were recorded using an inside-out patch configuration. The intracellular face was initially exposed to a zero-Ca^2+^ bath solution, and subsequently to bath solutions with 0.1 μM Ca^2+^. Currents were recorded by repetitive 1-s-voltage ramps from −100 mV to + 100 mV from a holding potential of 0 mV. One minute after switching of bath solutions, ten sweeps with a 1-s interval were recorded at a series of concentrations of the compounds in the presence of 0.1 μM Ca^2+^. The maximal SK2 current in response to 10 μM Ca^2+^ was then recorded before the integrity of the patch was examined by switching the bath solution back to the zero-Ca^2+^ buffer. Data from patches, which did not show significant changes in the seal resistance after solution changes, were used for further analysis. To construct the dose-dependent potentiation of channel activities, the current amplitudes at −90 mV in response to various concentrations of the compound were normalized to that obtained at maximal concentration of that compound. The normalized currents were plotted as a function of the concentrations of each compound. EC_50_ values and Hill coefficients were determined by fitting the data points to a standard dose–response curve (*Y* = 100/(1 + (X/EC_50_)^ −Hill)). All data are presented in mean ± s.e.m. The Student’s *t*-test was used for data comparison.

### Protein crystallization and structure determination

The protein complex consisting of CaM and the SK2 channel fragment (R395–Q486 from rat SK2-a channel) was purified as described in our previous papers^48,49,60^. Briefly, rat CaM was introduced into the pET28b(+) vector (Novagen) and expressed in Rosetta-2 *E. Coli*. cells (Novagen). The CaM protein was purified using a low substitution phenyl sepharose column (GE Healthcare). The SK2 channel fragment was also introduced into the pET28b(+) vector (Novagen) and the codons have been optimized to improve expression of this His-tagged protein in *E. Coli*. This His-tagged protein was expressed and purified using a Ni-NTA column. The purified SK2 channel fragment was mixed slowly with the purified CaM in the presence of Ca^2+^ to form a complex, followed by purification with a gel filtration column (GE Healthcare) pre-equilibrated in a solution with 10 mM Tris-HCl, 50 mM NaCl, and 10 mM CaCl_2_ (pH 7.5). The purified protein complex was concentrated to about 1 mM and then set up for crystallization. Protein crystals of the protein complex were grown in sitting drops by vapour diffusion at 20 °C. The complex (1 mM) was mixed in a 1:1 ratio with the reservoir solution, which consists of 1.5 M Li_2_SO_4_, 0.6 M (NH_4_)_2_SO_4_, 0.1 M sodium citrate, pH 5.8. Monoclinic crystals usually grew within 3 weeks. These preformed protein crystals were then incubated with SK channel modulators at their saturating concentrations for ~1 month (SKS-11) and ~5 months (SKS-14), respectively. The soaked protein crystals were flash-cooled in liquid nitrogen for data collection, after a brief transfer to a suitable cryoprotectant (25% glycerol with the reservoir solution saturated with SKS-11 or SKS-14). X-ray diffraction data were collected from single crystals on the X-ray diffraction system (D8 Venture Diffraction System, Bruker AXS Inc.) at our home institute and processed using PROTEUM2 (Bruker AXS Inc.). Initial phases were determined by molecular replacement (MR) using PHASER from the CCP4 suite^61^. Our previously determined CaM/CaMBD complex structure (4J9Y) was used as a starting model to phase diffraction data. Solvent molecules were removed from the starting molecule before rigid body refinement. The crystallographic model was further constructed through iterative rounds of manual model-building using Coot^62^ and crystallographic refinement using REFMAC^61^ and PHENIX^63^. SKS-11 and SKS-14 were modeled respectively in a pocket at the CaM-channel interface based on strong electron density in difference Fourier maps and followed by successful refinement of the coordinates. The crystallographic statistics for data collection and model refinement are summarized in Supplementary Table 1. Structure graphics were created using PyMol (Schrödinger, LLC).

### Interaction Energy Calculation

The Discovery Studio 3.5 molecular modeling program (Accelrys Software Inc.) was used to conduct energy minimization and calculate the interaction energy between the compound and the protein complex. All structures were subjected to energy minimization using Smart Minimizer algorithm (2000 steps), and Generalized Born (GB) implicit solvent model using the CHARMM force field. Interaction energies between the compound and the protein complex were calculated using a distance dependent dielectric constant (ε = 2r) implicit solvent model. The decomposed interaction energy contribution of residues to the total interaction energy was analyzed to identify the critical interacting amino acid residues in the protein complex.

### Data availability

The structure coordinates have been deposited in the Protein Data Bank under accession codes 5WBX and 5WC5.

## Electronic supplementary material

Supplementary Info: Structural insights into the potency of SK channel positive modulators
