# Supplementary material for: Structural insights into the potency of SK channel positive modulators
Source: Sci Rep. 2017 Dec 7;7:17178. doi: 10.1038/s41598-017-16607-8 (PMC5719431; doi:10.1038/s41598-017-16607-8)
Supplement: Supplementary file 1 — Supplementary Info: Structural insights into the potency of SK channel positive modulators [file 41598_2017_16607_MOESM1_ESM.pdf]

Supplementary Information

Structural insights into the potency of SK channel positive modulators

Young-Woo Nam [1], Razan Orfali [1], Tingting Liu [2,3], Kunqian Yu [2,3], Meng Cui [4], Heike Wulff [5], Miao Zhang [1,*]

[1] Department of Biomedical and Pharmaceutical Sciences & Structural Biology Research Center, Chapman University School of Pharmacy, Irvine, CA, 92618 , USA

[2] State Key Laboratory of Drug Research, Shanghai Institute of Materia Medica, Chinese Academy of Sciences, Shanghai, 201203, China

[3] University of Chinese Academy of Sciences, Beijing, 100049, China

[4] Department of Pharmaceutical Sciences, Northeastern University School of Pharmacy, Boston, MA, 02115, USA

[5] Department of Pharmacology, School of Medicine, University of California, Davis, CA, 95616, USA

[*] Correspondence should be addressed to M.Z. (zhang@chapman.edu)

Running title: Positive modulation of SK channels

Email: zhang@chapman.edu
Phone: 1-714-516-5478
Department of Biomedical and Pharmaceutical Sciences
Chapman University School of Pharmacy
9501 Jeronimo Road
Irvine, CA 92618

Table 1. Crystallographic statistics

**Data Collection**[a]		
	**CaM-CaMBD2-a with SKS-11**	**CaM-CaMBD2-a with SKS-14**
Space Group<br>Unit Cell Dimensions	C2<br>a=77.3 Å, b=66.1 Å, c=65.4 Å<br>α=90.0°, β=93.8°, γ=90.0°	C2<br>a=77.0 Å, b=66.0 Å, c=65.3 Å<br>α=90.0°, β=93.8°, γ=90.0°
Wavelength (Å)	1.3148	1.3424
Resolution range (Å)	26.84–1.9 (1.95-1.88)	27.86-2.30 (2.38-2.30)
Completeness (%)	98.4 (84.4)	99.6 (99.9)
Total Observations	165,310 (4,536)	95,275 (8,569)
Unique Observations	25,882 (1,568)	14,551 (1,431)
Mean Redundancy	6.4 (2.9)	6.5 (6.0)
Mean I/σ(I)	14.9 (3.2)	19.9 (8.7)
R_{merge}[b]	0.073 (0.368)	0.057 (0.156)
R_{pim}[c]	0.029 (0.245)	0.024 (0.068)
**Model Refinement**[a]		
Resolution Range (Å)	25.79-1.9 (1.97-1.9)	25.69-2.3 (2.38-2.3)
No. reflections	24,969 (2,043)	14,509 (1,424)
R_{work}[d]	0.188 (0.307)	0.196 (0.28)
R_{free}[d]	0.242 (0.398)	0.248 (0.36)
No. atoms / Avg. B (Å^2)	2,197/32.7	2,157/34.8
protein	1,931/31.4	1,931/34.0
calcium ions	2/35.2	2/47.9
solvent	218/38.7	175/34.6
ligand	48/56.1	51/64.9
Phi/Psi angles favored (%) / outliers (#)	99.6/0.4	99.6/0.4
r.m.s.d. bond angles (°)	1.1	1.5
r.m.s.d. bond lengths (Å)	0.007	0.008

[a] Values in parentheses refer to data in the highest resolution shell.

[b] $R_{merge} = \sum_{hkl}\sum_j |I_j - \langle I\rangle| / \sum_{hkl}\sum_j I_j$. $\langle I\rangle$ is the mean intensity of *j* observations of reflection *hkl* and its symmetry equivalents.

[c] R_{pim} (precision-indicating merge) = $\sum_{hkl} (1/n_{hkl} - 1)^{1/2} \sum_j |I_j - \langle I\rangle| / \sum_{hkl}\sum_j I_j$. *n* is the number of observations of reflection hkl.

[d] $R_{cryst} = \sum_{hkl} |F_{obs} - kF_{calc}| / \sum_{hkl} |F_{obs}|$. $R_{free} = R_{cryst}$ for 5% of reflections excluded from crystallographic refinement.

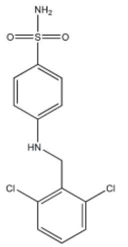
AN-465/42833836

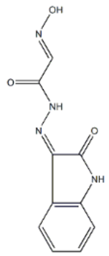
AG-205/13459166

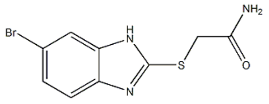
AG-670/11417235

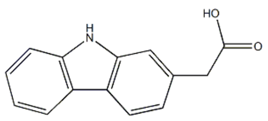
AP-124/40904377

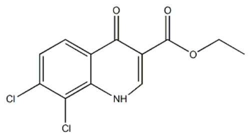
AF-399/13045008

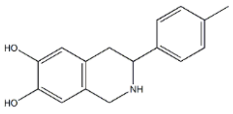
AE-641/00348017

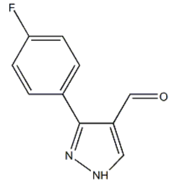
AK-968/13779224

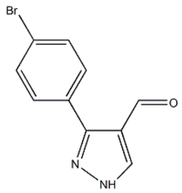
AK-968/11821765

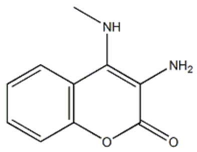
AH-262/31957064

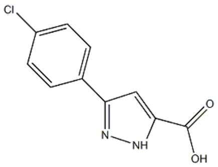
AG-690/14009300

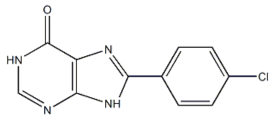
AC-907/34132054

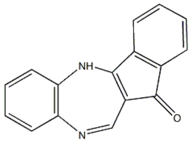
AG-205/07760007

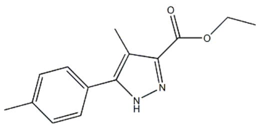
AR-360/42760872

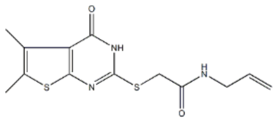
AF-399/37305025

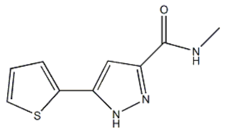
AN-329/43449444

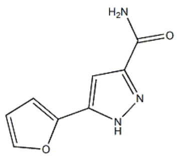
AN-329/43449442

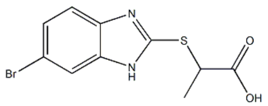
AG-670/40741590

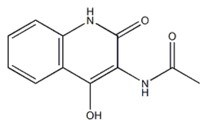
AG-690/34157012

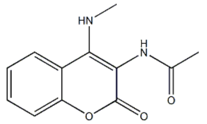
AG-690/12844897

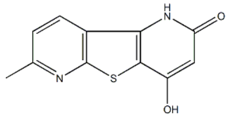
AM-807/25050039

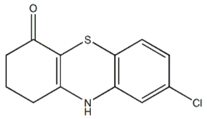
AH-262/32928006

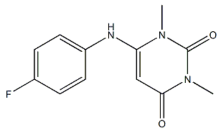
AI-204/31710042

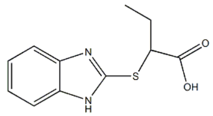
AR-422/41337951

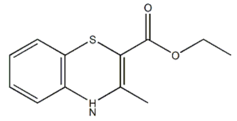
AG-690/13507288

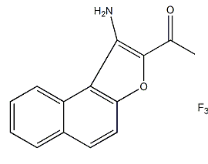
AP-147/15639031

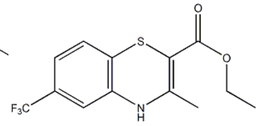
AG-205/14151044

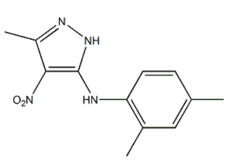
AG-690/33775048

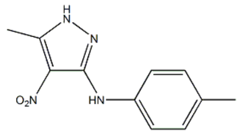
AG-690/10484061

Figure S1. The inactive compounds tested experimentally. Chemical structures and Specs ID (www.specs.net) of the 28 compounds tested inactive are shown.

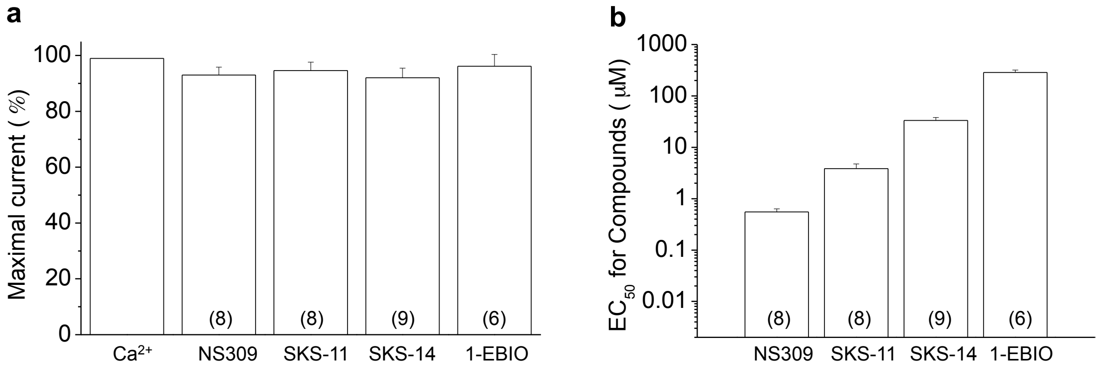

Figure S2. The efficacy and potency of NS309, SKS-11, SKS-14 and 1-EBIO. (a) The maximal responses to compounds are normalized by the SK2 current induced by saturating 10 μM Ca^{2+}. (b) The potency (EC_{50}) of the four compounds in potentiating the SK2 current is different from each other. All data are presented in mean ± s.e.m. The numbers of experiments are shown in the parentheses.

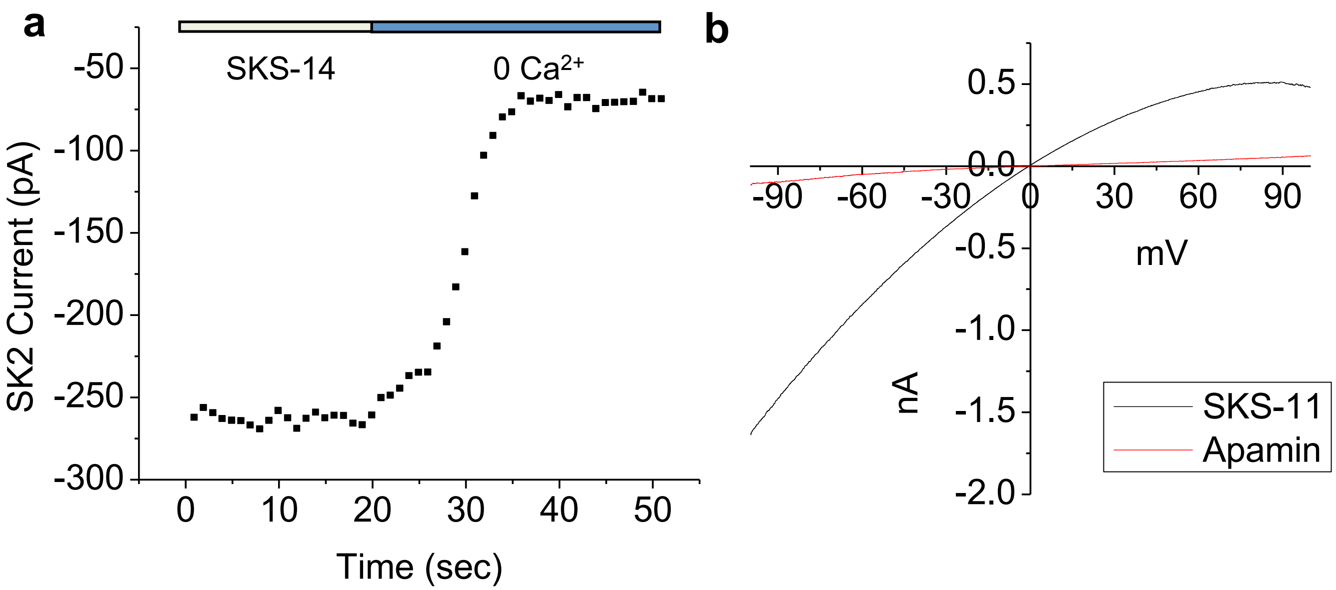

Figure S3. Control experiments of SK2 current. (a) In inside-out patch recordings, 0 Ca^{2+} inhibited the current induced by SKS-14 (100 μM) in the presence of 0.1 μM Ca^{2+}. (b) In whole-cell recordings, apamin inhibited the current induced by SKS-11 (30 μM).
